# Benefit–Risk Assessment of the French Surveillance Protocol of Apparently Healthy Biting Dogs and Cats for Human Rabies Prevention

**DOI:** 10.3390/vetsci8070132

**Published:** 2021-07-13

**Authors:** Guillaume Crozet, Tiffany Charmet, Florence Cliquet, Emmanuelle Robardet, Barbara Dufour, Julie Rivière

**Affiliations:** 1 Laboratoire de Santé Animale USC EPIMAI, Anses, Ecole Nationale Vétérinaire d’Alfort, F-94700 Maisons-Alfort, France; tiffany.charmet@pasteur.fr (T.C.); barbara.dufour@vet-alfort.fr (B.D.); julie.riviere@vet-alfort.fr (J.R.); 2 Emerging Disease Epidemiology Unit, Institut Pasteur, F-75015 Paris, France; 3 Nancy Laboratory for Rabies and Wildlife, Anses, F-54220 Malzéville, France; florence.cliquet@anses.fr (F.C.); emmanuelle.robardet@anses.fr (E.R.)

**Keywords:** rabies, bite, dog, cat, surveillance, scenario-tree model, benefit–risk

## Abstract

In France, apparently healthy dogs and cats that bite humans must undergo an observation period of 15 days with three veterinary visits to ascertain that they remain healthy, indicating that no zoonotic transmission of rabies virus occurred via salivary presymptomatic excretion. This surveillance protocol is mandatory for all pets that have bitten humans, despite France’s rabies-free status in non-flying mammals (i.e., a very low rabies risk). In this context, we aimed to perform a benefit–risk assessment of the existing regulatory surveillance protocol of apparently healthy biting animals, as well as alternative surveillance protocols. A scenario-tree modelling approach was used to consider the possible successions of events between a dog or cat bite and a human death attributed to either rabies or to lethal harm associated with the surveillance protocol (e.g., lethal traffic accidents when traveling to veterinary clinics or anti-rabies centers). The results demonstrated that the current French surveillance protocol was not beneficial, as more deaths were generated (traffic accidents) than avoided (by prompt post-exposure prophylaxis administration). We showed here that less stringent risk-based surveillance could prove more appropriate in a French context. The results in this study could allow policy-makers to update and optimize rabies management legislation.

## 1. Introduction

Animal bites and more specifically dog and cat bites are a frequent occurrence throughout the world, and France is no exception [1,2,3,4,5,6]. Due to a large pet population of 6,950,000 dogs and 13,500,000 cats [7], French citizens are routinely exposed to these adverse events that can have traumatic and/or psychological consequences [4]. Dog and cat bites also play a role in zoonotic pathogen transmission [8,9,10]. Indeed, dog and cat commensal oral micro-flora often lead to polymicrobial contaminations of bite wounds with certain microorganisms associated with systemic manifestations, like *Pasteurella* spp., *Neisseria animaloris* or *Capnocytophaga carnimorsus* [10]. Rabies Virus (RABV) can also be transmitted to humans by dog and cat bites. RABV infections lead to a 100% lethal zoonosis once symptoms appear and have major public health implications with an estimation of 60,000 annual human deaths reported around the world, with dog bites accounting for 99% of these human cases [11].

In 2001, France was declared rabies-free in non-flying mammals according to the World Animal Health Organization’s (OIE) definition. Since this declaration, 14 animal rabies cases due to RABV infection were reported: 12 were related to importations of pets from rabies enzootic countries or travel to such areas, and two were related to secondary indigenous transmissions. The rabies control strategy in France for domestic dogs and cats relies on pet importation regulations, such as proper pet identification methods, vaccination, serological testing, and border controls for pets entering the French territory [12] as well as the implementation of specific response and containment measures for suspected and confirmed cases of rabies [13].

A surveillance protocol of apparently healthy animals that have bitten humans has also been put in place in order to ascertain that presymptomatic salivary excretion of the rabies virus does not lead to an unaccounted transmission event. This surveillance protocol is based on individual declarations of bites to the appropriate veterinary services, and on the subsequent two-week surveillance period for the biting animal, during which three visits for veterinary clinical examination are performed (on the day of the bite or within 24 h, bite + 7 days, and bite + 15 days) [14]. 

According to a recent study, this surveillance protocol appears to be largely unfamiliar to pet-owners, and moderately accepted by veterinarians [15]. Indeed, this surveillance protocol concerns all biting cats and dogs regardless of their individual risk-level for having acquired the rabies virus, thus, generating a high burden of time and effort for pet owners and veterinarians, while the rabies infection probability remains low for the majority of biting pets [16].

French rabies management legislation could, thus, be evaluated and updated using a benefit–risk approach, taking into account the two decades of rabies-free status in the country [17]. Other authors have already used a benefit–risk approach in the context of rabies risk: for example, Gunther et al. (2008) estimated that the probability of contracting rabies abroad for travelers from Germany was comparable to the death probability by traffic accident on a 6-km car trip (i.e., extremely low) [18]. 

Ribadeau-Dumas et al. (2015) showed that, in France, systematic rabies post-exposure prophylaxis (PEP) administration to bite victims when the biting animal was not observable could be detrimental by generating a higher probability of human death (linked to traffic accidents when commuting to an anti-rabies center) when compared to no action [19]. Similarly, there is a chance that the existing rabies surveillance protocol for biting animals in France is detrimental, generating a higher probability of human death (due to traffic accidents, for example) when compared to the reduction of overall rabies death probability (by administering PEP to people bitten by a rabies-infected pet).

Moreover, other European countries with a rabies epidemiological context similar to France have decompressed legislation to allow for less stringent measures with regards to the surveillance of apparently healthy biting animals. For example, for bites occurring in rabies-free European countries, such as Belgium [20], Switzerland [21], and the United Kingdom (UK) [22], an observation period is recommended only if the biting animal was recently imported (i.e., at-risk for rabies), or if the bite occurred in an area where a case in non-flying mammals had been recently detected. 

The surveillance protocol consists of observing the biting animal for a period of 10 days in Switzerland and Belgium, and 15 days in the UK. The implementation of such observation periods can justify postponing the beginning of the PEP administration, as proposed in Switzerland and in the UK [21,22]. More generally, the World Health Organization (WHO) also recommends a 10-day observation period for biting animals in order to either discontinue PEP administration (if started promptly after the bite event) or to delay the start of PEP administration in certain cases. 

Nevertheless, the WHO recommendations apply to countries enzootic for rabies in non-flying mammals. In areas declared free of rabies for all non-flying mammals, PEP administration is not recommended for the majority of dog and cat bites (although this decision should follow a proper risk assessment by a health professional) [23]. These discrepancies between countries with similar epidemiological contexts for rabies further highlight the necessity for an assessment of the surveillance protocols in France.

In this framework, we aimed to conduct a benefit–risk assessment of the current French surveillance protocol of apparently health biting dogs and cats for human rabies prevention. We also aimed to conduct benefit–risk assessments of less-stringent alternative surveillance protocols that could profit from a wider acceptance by both pet owners and veterinarians in accordance with the French epidemiological context for rabies.

## 2. Materials and Methods

### 2.1. Surveillance Protocol Modelling

For the purpose of this benefit–risk assessment, a scenario-tree modelling approach was adopted. Several surveillance protocols were modelled and the specificities of each protocol are presented below in the appropriate parts. We present here the general principles applied to all protocols:

For all investigated protocols, the possible successions of events from a cat or dog bite to a human death were considered within an epidemiological window of 15 days following the bite event. Models were limited to a 15-day period after the bite by an apparently healthy dog or cat, as this corresponded to the period during which PEP could be started (based on a maximum observed period of presymptomatic salivary RABV excretion by a dog [24] plus a two-day security period). Human deaths modelled in this framework were either due to RABV infections or due to lethal harm associated with the surveillance protocol. For the latter category, lethal traffic accidents occurring during the commute to a veterinary clinic or anti-rabies center were considered, as they were the only events (with RABV infections) that possibly led to human deaths in the context of the surveillance protocol.

In the scenario-trees, the pet owner and veterinarian compliance was modelled. Owners could not attend veterinary visits for several scenarios: (i) non-started surveillance protocol (i.e., bite event not declared to a veterinarian); (ii) early termination of the surveillance protocol (secondary veterinary visit(s), if applicable, not carried out). Owners could also neglect to declare the manifestation of clinical signs in their pet while undergoing at-home observation. Similarly, the veterinarian could start or not start a surveillance protocol after a bite event declaration by a pet owner.

Spontaneous declarations of neurological clinical signs in the biting cat/dog by the pet owner were also considered even if the declaration was made outside of the surveillance protocol. Several scenarios for spontaneous declarations were considered (limited by the epidemiological window of 15-days post-bite): (i) declaration after the surveillance period; (ii) declaration after an early termination of the surveillance protocol; and (iii) declaration in cases where no surveillance was started.

We considered that, in the event of rabies clinical sign manifestation and subsequent declaration to the veterinarian by the dog/cat owner, and in turn to the French veterinary services by the veterinarian, the bitten person would promptly seek PEP (Zagreb regimen with three visits to an anti-rabies center). The declaration of clinical signs by the veterinarian to veterinary services was considered to be systematic if they occurred during the surveillance period or after an early termination. 

In the case of non-started surveillance protocol, the declaration of a rabies suspicion by the veterinarian (following the owner declaration to the veterinarian) relied on the veterinarian’s ability to suspect rabies. No lethal harm associated with PEP administration was implemented in our models as it is apparently safe [23]. PEP was also considered to be 100% effective, as instances of failure during proper administration appear to be extremely rare [25].

#### 2.1.1. Current Surveillance Protocol “3V”

**Figure 1:**
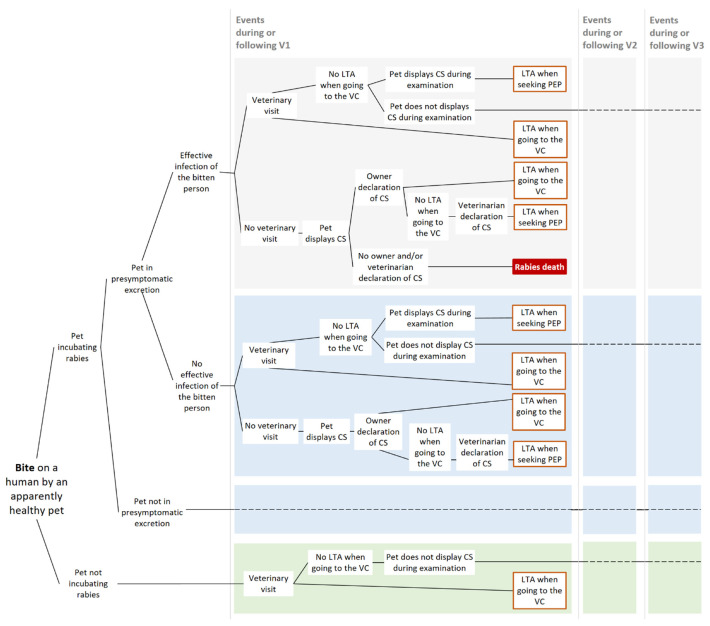
Scenario-tree modelling of the current surveillance protocol (“3V”) of apparently healthy biting dogs and cats. Only pathways leading to a human death within an epidemiological window of 15 days post-bite are represented. V1 (−2 and −3): veterinary visit 1 (−2 and −3) on day 1 (7 and 15), LTA: lethal traffic accident; VC: veterinary clinic; and CS: clinical signs. Dashed lines indicate that the same events occurring in the box of same color are repeated, but the parameterization can be different (see Section 2.2).

In France, the surveillance of apparently healthy biting animals relies on three visits to a veterinarian for clinical examination of the animal in order to ensure that no clinical signs of rabies manifest during the 15-day period following the bite event [14]. All possible pathways from a dog/cat bite event to a human death for the current French surveillance protocol are summarized in Figure 1.

#### 2.1.2. Absence of Surveillance Protocol “M0”

**Figure 2:**
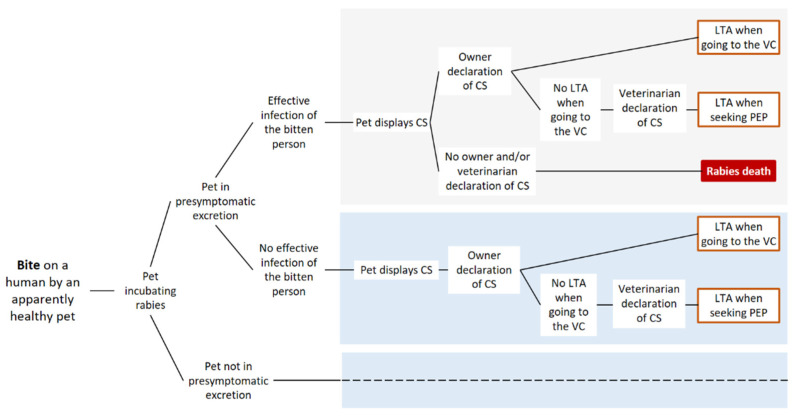
Scenario-tree modelling of the absence of surveillance protocol (“M0”) of apparently healthy biting dogs and cats. Only pathways leading to a human death within an epidemiological window of 15 days post-bite are represented. LTA: lethal traffic accident; VC: veterinary clinic; and CS: clinical signs. Dashed lines indicate that the same events occurring in the box of same color are repeated, but the parameterization can be different (see Section 2.2).

In the absence of defined surveillance protocol, a bitten person would seek PEP only if the cat/dog displaying clinical signs of rabies was brought to a veterinarian, identified, and subsequently declared as being suspected of rabies. The possible successions of events from a bite to a human death (within the epidemiological window of 15 days post-bite) in the absence of a specific surveillance protocol are represented in Figure 2.

#### 2.1.3. Less Stringent Alternative Surveillance Protocol “1V10D”

**Figure 3:**
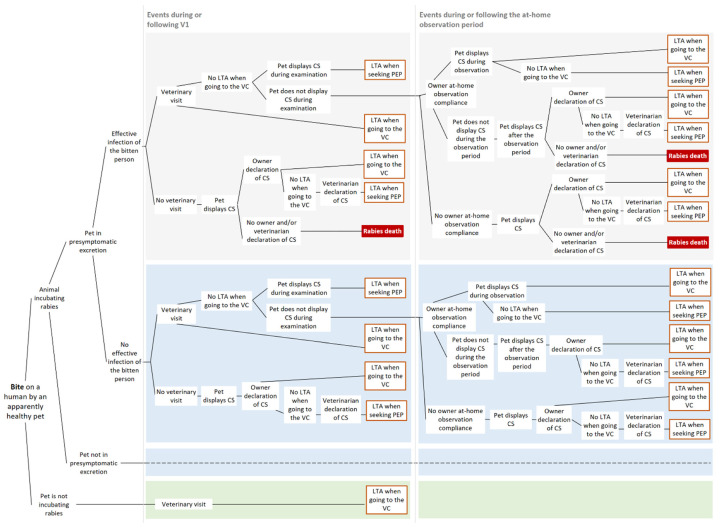
Scenario-tree modelling of the less stringent alternative surveillance protocol (“1V10D”) of apparently healthy biting dogs and cats. Only pathways leading to a human death within an epidemiological window of 15 days post-bite are represented. V1: veterinary visit 1 on day 1, LTA: lethal traffic accident; VC: veterinary clinic; and CS: clinical signs. Dashed lines indicate that the same events occurring in the box of same color are repeated, but the parameterization can be different (see Section 2.2).

To investigate the effect of a less stringent surveillance protocol (i.e., with a reduced number of veterinary visits), we chose to model a surveillance protocol with only one veterinary visit on the day of the bite event, followed by a 10-day at-home observation period of the animal by the owner. This alternative protocol supposed that at-home surveillance compliance was verified at the end of the 10-day period by a veterinarian (via a follow-up phone call, for example) in order to certify the absence of clinical signs and the survival of the dog/cat responsible for the biting-event. The successions of events from a bite to a human death (within the epidemiological window of 15 days post-bite) for this less stringent surveillance protocol are represented in Figure 3.

#### 2.1.4. Risk-Based Alternative Surveillance Protocols “S_3V_1V10D”, “S_3V_M0”, and “S_1V10D_M0”

Here, we defined a pet population selection at-risk for rabies corresponding to: (i) pets having travelled outside the EU in the previous year (legally or illegally); (ii) pets having travelled illegally within the EU (and outside France) in the previous year; (iii) all newly imported pets in the previous year. We then proposed differential management of biting animals considering this “at-risk”/“non-at-risk” dichotomy. Three alternative surveillance protocols combining surveillance strategies based on this classification were investigated (Table 1).

### 2.2. Parameterization

When possible, parameters were modelled by distributions to account for uncertainty associated to biological variability and/or lack of data.

#### 2.2.1. Probability of Rabies Infection of French Pets

The annual rabies incidence was modelled by a Gamma distribution assuming that these cases occurred through a Poisson process and by using Bayesian inference with a non-informative prior [26]. To obtain the probability of one pet being infected on one day (i.e., the instantaneous prevalence of pets incubating rabies), we multiplied the annual incidence by $\frac{\mathrm{mean}\left(Inc\right)}{365.24}$ or $\frac{\mathrm{mean}\left(Inc_{Fr}\right)}{365.24}$ where Inc is the rabies incubation period and where $Inc_{Fr}$ is the rabies incubation period occurring in France for imported animals in days, (see below for more details about incubation periods). This assumed a homogenous repartition of rabies cases over time.

Considering that 11 dog rabies cases infected while abroad, 2 dog rabies cases infected while in France (secondary infections), one cat rabies case infected while abroad, and no cat rabies cases infected while in France were declared over a 20-year period (2001–2020) in France [27,28,29], we defined the following distributions for the instantaneous case numbers (ICN) of non-indigenous dog rabies ($ICN_{dog,nind}$), ICN of indigenous dog rabies ($ICN_{dog,ind}$), ICN of non-indigenous cat rabies ($ICN_{cat,nind})$, and ICN of indigenous cat rabies ($ICN_{cat,ind})$:

$$ICN_{dog,nind}=\mathrm{Gamma}\left(11;\frac{1}{20}\right)\times \frac{\mathrm{mean}\left(Inc_{Fr}\right)}{365.24}\;$$

$$ICN_{dog,ind}=\mathrm{Gamma}\left(2;\frac{1}{20}\right)\times \frac{\mathrm{mean}\left(Inc\right)}{365.24}\;$$

$$ICN_{cat,nind}=\mathrm{Gamma}\left(1;\frac{1}{20}\right)\times \frac{\mathrm{mean}\left(Inc_{Fr}\right)}{365.24}\;$$

$$ICN_{cat,ind}=\mathrm{Gamma}\left(0;\;\frac{1}{20}\right)\times \frac{\mathrm{mean}\left(Inc\right)}{365.24}\;$$

The total ICN values for dogs and cats were then defined as: $ICN_{dog}=ICN_{dog,nind}+ICN_{dog,ind}$ and $ICN_{cat}=ICN_{cat,nind}+ICN_{cat,ind}$.

To obtain the corresponding instantaneous prevalences, $Pr_{dog,nind}$, $Pr_{dog,ind}$, $Pr_{cat,nind}$, $Pr_{cat,ind}$, $Pr_{dog}$ and $Pr_{cat}$, the ICN values were divided by the appropriate population sizes, which were, respectively, $POP_{dog}\times P_{dogAR}$, $POP_{dog}\times (1-P_{dogAR})$, $POP_{cat}\times P_{catAR}$, $POP_{cat}\times (1-P_{catAR})$, $POP_{dog}$, and $POP_{cat}$. We used FEDIAF 2018 data for the French dog and cat population sizes, with $POP_{dog}$ = 6,950,000 and $POP_{cat}\;$= 13,500,000 [7]. $P_{dogAR}$ and $P_{catAR}$ corresponded to the proportion of dogs or cats being “at-risk” for introducing rabies (see below, Section 2.2.6, for the definition of this parameter).

#### 2.2.2. Probability of a Pet Being in Presymptomatic Excretion Period of Rabies Virus While Incubating Rabies

Rabies transmission by apparently healthy pets incubating rabies can only occur during the presymptomatic excretion period. To obtain the probability of a pet being in presymptomatic excretion, we divided the presymptomatic excretion period by the incubation period. Since risk-based surveillance protocols distinguish between indigenous and non-indigenous rabies cases (i.e., linked to pet importation or travel), we employ this dichotomy throughout this paragraph.

The rabies incubation distribution was obtained by gathering data regarding the incubation from natural dog and cat infections [29,30,31]. Quarantine data were included, even if they corresponded to truncated incubation periods, since it contributed to increase the variance even if a conservative 6-day period was added (corresponding to the minimum incubation period observed in the other references) to account for the fact that these animals were already incubating rabies when selected to be observed in quarantine. A lognormal distribution was then fitted by the maximum likelihood estimation (MLE): with a mean of 36.8 days and a standard deviation (SD) of 40.4 days.

$$Inc=\mathrm{Lognormal}\left(3.21;\;0.89\right)$$

For animals that were imported while incubating rabies, the incubation period occurring in France was estimated as follows: we randomly selected an importation day inside the Inc distribution, and this period (between infection and importation) was subtracted from the Inc distribution to provide $Inc_{Fr}$ (mean of 18.6 days and SD of 25.6 days).

In order to obtain a distribution for the presymptomatic excretion period, we gathered reports of experimental rabies infections providing RABV excretion periods [24,32,33] and fitted a lognormal distribution by MLE:

$$Exc=\mathrm{Lognormal}\left(0.56;\;0.68\right).$$

This distribution was then right-truncated so as not to exceed the incubation period. This distribution had a mean of 2.15 days and a SD of 1.56 days.

We also defined the presymptomatic excretion period occurring in France ($Exc_{Fr}$) for imported animals by randomly selecting an importation day within the distribution of the incubation period and by, then, subtracting the presymptomatic excretion period occurring abroad if the importation day was during the presymptomatic excretion period. $Exc_{Fr}$ had a mean of 1.97 days and a SD of 1.46 days.

The probabilities $P_{Exc,ind}$ and $P_{Exc,nind}$ of an animal incubating rabies being in presymptomatic excretion for indigenous and non-indigenous cases, respectively, were then defined as:

$$P_{Exc,ind}=\frac{Exc}{Inc}\;(\mathrm{mean}=0.12;\;\mathrm{SD}=0.15)\;\mathrm{and}\;P_{Exc,nind}=\frac{Exc_{Fr}}{Inc_{Fr}}\;(\mathrm{mean}=0.32;\;\mathrm{SD}=0.33).$$

We also defined $\;P_{Exc}$ for scenarios not distinguishing between indigenous and non-indigenous rabies cases (i.e., non-risk-based surveillance protocols) as a weighted mean of $P_{Exc,\;ind}$ and $P_{Exc,n\;ind}$ with weights based on the indigenous ICN and non-indigenous ICN presented above.

#### 2.2.3. Probability of a Rabies Infected Pet Displaying Clinical Signs on a Given Day after the Bite

For an asymptomatic dog or cat that has bitten a human, the probability of displaying clinical signs on a given day after the bite depended on the date of the bite (within the incubation period) and on the incubation period length. In our scenario-tree models (Figure 1, Figure 2 and Figure 3), we distinguished between infected animals in the presymptomatic excretion period when biting (i.e., in late incubation) and those before the presymptomatic excretion period. Similarly to the previous paragraph, we used the dichotomy between indigenous and non-indigenous rabies cases.

First, for those in the presymptomatic excretion period, we randomly selected a biting day within the presymptomatic excretion period distribution to define the RExc and $RExc_{Fr}$, distributions of the remaining presymptomatic excretion periods at the time of the bite. The probabilities $P_{CSexc,ind,j}$ and $P_{CSexc,nind,j}$ of displaying clinical signs on j days after the bite, for indigenous and non-indigenous rabies cases, respectively, were, thus, defined as:

$$P_{CSexc,ind,j}=P\left(RExc\geq j\right)\;\mathrm{and}\;P_{CSexc,nind,j}=P\left(RExc_{Fr}\geq j\right).$$

**Figure 4:**
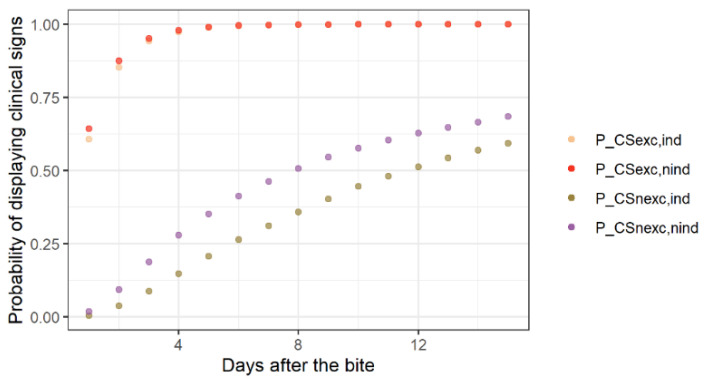
The probability of a rabies-incubating dog or cat displaying clinical signs on a given day after a bite.

We followed the same procedure for animals incubating rabies and biting before the presymptomatic excretion period. The probabilities $P_{CSnexc,ind,j}$ and $P_{CSnexc,nind,j}$ were defined as:

$$P_{CSnexc,ind,j}=P\left(RInc\geq j\right)\;\mathrm{and}\;P_{CSnexc,nind,j}=P\left(RInc_{Fr}\geq j\right)$$

where RInc and $RInc_{Fr}\;$ are the distributions of the remaining incubation periods after a bite (excluding period of presymptomatic excretion). The values of these probabilities for different values of j are presented in Figure 4.

We also defined $P_{CSexc,j}$ and $P_{CSnexc,j}$ for models not distinguishing between indigenous and non-indigenous rabies cases (i.e., non-risk-based surveillance protocols) as the weighted mean of $P_{CSexc,ind,j}$ and $P_{CSexc,nind,j}$, $P_{CSnexc,ind,j}$, and $P_{CSnexc,nind,j}$, respectively, with weights based on the indigenous ICN and non-indigenous ICN presented above.

#### 2.2.4. Probability of Human RABV-Infection after a Bite by an Infectious Animal

We used data provided by Shim et al. (2009) [34] to quantify the probability of human infection and death after a bite by a rabid animal (without PEP administration) considering the different bite anatomic sites (called $P_{D}$ below). The probability for a bite to occur at a specific anatomic site (called $P_{MORS}$ below) was provided by a study of *Santé Publique France* [35]. This study only dealt with dog bites, and we used same data for cat bites in our models since no other data was available. 

Each of these probabilities was defined as a Beta distribution using Bayesian inference and assuming a Bernoulli process (with non-informative priors) as follows [26]: $Beta\left(a+1;\;b-a+1\right)$ where a corresponded to the number of “successes” (e.g., the number of rabies deaths following bites by rabid animals), and b corresponded to the total number of events (e.g., the total number of bites by rabid animals). Thus, $P_{inf}$, the probability for a human of being infected and dying after a bite by a rabid animal, was defined as:

$$P_{inf\;}=P_{MORSarm}\times P_{Darm}+P_{MORSleg}\times P_{Dleg}+P_{MORShead}\times P_{Dhead}+P_{MORStrunc}\times P_{Dtrunc}\;,$$

with $P_{MORSarm}=\mathrm{Beta}\left(242+1;\;485-242+1\right)$; $P_{MORSleg}=\mathrm{Beta}\left(97+1;\;485-97+1\right)$; $P_{MORShead}=\mathrm{Beta}\left(116+1;\;485-116+1\right)$; $P_{MORStrunc}=\mathrm{Beta}\left(29+1;\;485-29+1\right)$ and $P_{Darm}=\mathrm{Beta}\left(8+1;\;36-8+1\right)$; $P_{Dleg}=\mathrm{Beta}\left(6+1;\;51-6+1\right)$; $P_{Dhead}=\mathrm{Beta}\left(6+1;\;11-6+1\right)$; $P_{Dtrunc}=\mathrm{Beta}\left(1+1;\;11-1+1\right)$.

#### 2.2.5. Probability of Lethal Harm Associated with the Surveillance Protocols

As presented above, we considered lethal traffic accidents as a lethal harm associated with the surveillance protocols. Such accidents can occur when commuting to a veterinary clinic or when commuting to an anti-rabies center for PEP administration.

In France, the probability of death by traffic accident per kilometer for the year 2019 ($P_{LTAkm})$ was 3.08 × 10^−9^ [36,37]. The distribution of the distance to a veterinary clinic for a pet owner was evaluated by randomly sampling French postal addresses (*n* = 448) in a national file [38] and searching for distances by car to the two closest veterinary clinics on Google Map™. The distribution was then defined by randomly selecting one of the two veterinary clinics (to model preferences of the owners who do not always go to the closest veterinary clinic) and by fitting a Gamma distribution on this generated dataset by MLE. This distance was then multiplied by 2 to have the distribution for a round-trip:

$$Dist_{VC}=\mathrm{Gamma}\left(\mathrm{shape}\;=1.37;\;\mathrm{rate}\;=0.18\right)\;\times \;2.$$

The distance travelled for a complete PEP was provided by Ribadeau-Dumas et al. (2015) [19], and a non-parametric PERT distribution was used:

$$Dist_{PEP}=\mathrm{PERT}\left(\min\;=0.6;\;\mathrm{mode}=180;\;\max=800\right).$$

Then, the probability of death per kilometer was multiplied by these distances to obtain the death probabilities during car journeys $P_{LTAclin}$ and $P_{LTAPEP}$:

$$P_{LTAclin}=Dist_{VC}\times P_{LTAkm}\;\mathrm{and}\;P_{LTAPEP}=Dist_{PEP}\times P_{LTAkm}.$$

#### 2.2.6. Probability of a Pet Being At-Risk

To obtain the probability for a pet to have travelled outside the EU (legally or illegally) in the previous year or travelled illegally outside France but within the EU, we used data published elsewhere [39] to fit the Beta distributions as described above. We used weighted data counts following the post-stratification process described in the above-mentioned study. For the number of newly imported pets in the previous year, we used I-CAD (*Identification des carnivores domestiques*) 2018 data corresponding to foreign microchips newly registered in France during 2018. These parameters are presented in Table 2.

The probability $P_{dogAR}$ and $P_{catAR}$ for a dog and a cat, respectively, to be at-risk were calculated as follows:

$$P_{dogAR}=P_{travnEU,dog}+P_{travillEU,dog}+P_{imp,dog}\;\mathrm{and}\;P_{catAR}=P_{travnEU,cat}+P_{travillEU,cat}+P_{imp,cat}.$$

#### 2.2.7. Compliance Parameters

In our models, pet owner and veterinarian compliance could be imperfect and was modelled with several probability distributions. The data sources included the results of a survey presented in Supplementary Material S1 and the authors’ hypotheses (completed with another data source [15]). These compliance distributions are presented in Table 3.

#### 2.2.8. Number of Dog and Cat Bites in France

In order to calculate numbers of human deaths (rabies deaths or lethal harm associated with surveillance protocols), bite incidences were required to be multiplied by human death probabilities. These bite incidences were obtained through the survey previously mentioned and described in Supplementary Material S1. The survey provided the annual number of bites per person and to obtain the annual number of bites in France, we applied the central limit theorem given the large number of bites occurring in France [26]:

$$N_{mors}=\text{Normal}\left(\mu_{mors}\times POP_{Fr};\;\sqrt{POP_{Fr}\;}\times SD_{mors}\right)$$

where $POP_{Fr}$ corresponded to the human French population with a value of 64,737,769 [40], $\mu_{mors}$ corresponded to the mean of the annual number of bites per person and took a value of 1.04 × 10^−2^ for dogs and 4.30 × 10^−2^ for cats, and $SD_{mors}$ corresponded to the standard deviation of the annual number of bites per person and took the value of 1.10 × 10^−1^ for dogs and the value of 2.47 × 10^−1^ for cats (Supplementary Material S1).

### 2.3. Mortality and Benefit–Risk Indicator

- Number of deaths associated with the surveillance protocols

For each surveillance protocol model, we calculated the occurrence probability of each branch by multiplying the probabilities of the events on the considered branch. Then, the probabilities of each type of death (i.e., rabies, traffic accident, or both) associated with the surveillance protocol following one bite were obtained by summing the branch probabilities leading to the type of death considered.

The death probabilities (after one bite) were then multiplied by the annual bite numbers $N_{mors,dog}$ and $N_{mors,cat}$ to obtain the numbers of deaths occurring within the surveillance protocols.

Details of the calculations to provide these numbers of deaths is presented in Supplementary Material S2.

- Benefit–risk indicator

In order to evaluate the benefit–risk of each surveillance protocol presented above we defined a Benefit–Risk Ratio (BRR) as the ratio of the avoided number of deaths due to the surveillance protocol over the generated number of deaths due to the surveillance protocol. The BBR was, thus, defined as:

$$\text{BRR}=\;\frac{\text{Rabies deaths in M}0-\text{Rabies death in ME}}{\text{Lethal traffic accident in ME}-\text{Lethal traffic accidents in M}0}$$

where M0 is the absence of surveillance protocol (see Section 2.1.2) and ME is the evaluated model. If the BRR > 1, ME can be judged as beneficial (and non-beneficial if BBR < 1).

We then performed 10,000 Monte-Carlo simulations to obtain distributions for numbers of deaths and BRR. For each new simulation, new parameter values were randomly drawn is the presented distributions. Simulations were run using R language, R Studio software, and the “mc2d” package [41,42,43]. Convergence was assessed graphically using the “converge” function of this package. MLE were performed using the “fitdistrplus” package [41].

### 2.4. Sensitivity Analyses

#### 2.4.1. Sensitivity Analyses Based on Spearman Rank Correlation

For each model, we estimated the Spearman correlation, a rank-based measure, to evaluate the association between the uncertainty of the BBR (the output variable of interest) and the uncertainty of each of all input variables. To perform these sensitivity analyses, the function “tornado” of the “mc2d” package was used [41].

#### 2.4.2. One-Way Sensitivity Analyses

To assess the impact of changes of certain input parameters of interest, we evaluated the BBR for ranges of values out of the bonds of the initial input distributions. Such sensitivity analyses were performed on the rabies incidence to illustrate the impact of the change of rabies epidemiology (e.g., the situation of other countries) and the distance to the veterinary clinic to illustrate the impact of the density of the veterinary clinic network. We also performed these analyses on the compliance parameters to assess the potential impact of communication influencing the awareness of rabies risk (for owners and/or veterinarians), and also because these parameters mostly relied on assumptions.

## 3. Results

### 3.1. Mortality and Benefit–Risk Ratios

**Figure 5:**
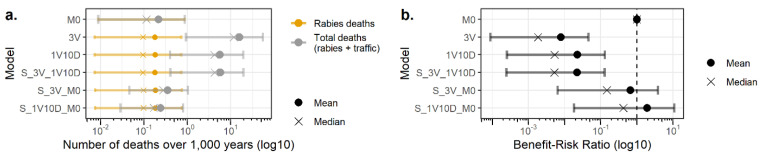
Number of human deaths (**a**) and the benefit–risk ratios (**b**) associated with the surveillance of apparently healthy biting dogs. Bars represent 95% confidence intervals. M0: absence of surveillance protocol; 3V: current French surveillance protocol with three veterinary visits within 15 days; 1V10D: less stringent alternative surveillance protocol relying on one veterinary visit and 10 days of observation at home; and S_3V_1V10D, S_3V_M0, and S_1V10D_M0: risk-based alternative surveillance protocols combining previous protocols (the first protocol of the name is applied to at-risk pets and the second protocol of the name is applied to non-at-risk pets).

**Figure 6:**
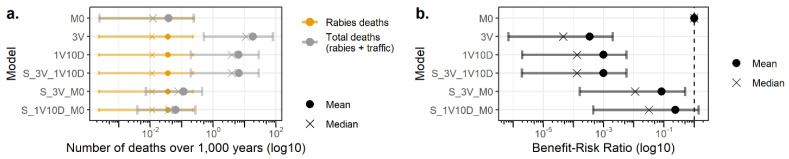
Number of human deaths (**a**) and the benefit–risk ratios (**b**) associated with the surveillance of apparently healthy biting cats. Bars represent 95% confidence intervals. M0: absence of surveillance protocol; 3V: current French surveillance protocol with three veterinary visits within 15 days; 1V10D: less stringent alternative surveillance protocol relying on one veterinary visit and 10 days of observation at home; and S_3V_1V10D, S_3V_M0, and S_1V10D_M0: risk-based alternative surveillance protocols combining previous protocols (the first protocol of the name is applied to at-risk pets and the second protocol of the name is applied to non-at-risk pets).

We evaluated the number of human deaths occurring in the context of the current and alternative surveillance protocols of apparently healthy biting animals and the BRR for these models. The results are presented in Figure 5 for dog bites and in Figure 6 for cat bites.

These results showed that all surveillance protocols of apparently healthy biting dogs contributed to decrease the mean number of human rabies deaths from 2.18 × 10^−1^ deaths/1000 years for the absence of surveillance protocol (M0) to 1.80 × 10^−1^ deaths/1000 years for the 3V, 1V10D, and S_3V_10D protocols and to 1.85 × 10^−1^ deaths/1000 years for the S_3V_M0 and S_1V10D_M0 surveillance protocols. For cat bites, the mean number of human rabies deaths decreased from 3.81 × 10^−2^ deaths/1000 years in the absence of surveillance protocol (M0) to 3.62 × 10^−2^ deaths/1000 years for the 3V, 1V10D, S_3V_1V10D, S_3V_M0, and S_1V10D_M0 surveillance protocols. 

Nonetheless, the mean total number of human deaths (including rabies deaths and traffic accident deaths) increased due to traffic accidents from 2.18 × 10^−1^ deaths/1000 years without surveillance protocol (M0) to 15.90, 5.66, 5.78, 3.52 × 10^−1^, and 2.43 × 10^−1^ deaths/1000 years for the 3V, 1V10D, S_3V_10D, S_3V_M0, and S_1V10D_M0, respectively, for dog bites. For cat bites, the mean total number of human deaths increased from 3.81 × 10^−2^ deaths/1000 years (M0) to 18.65, 6.52, 6.58, 1.16 × 10^−1^, and 6.40 × 10^−2^ deaths/1000 years for the 3V, 1V10D, S_3V_10D, S_3V_M0, and S_1V10D_M0, respectively.

The BBR values for alternative surveillance protocols were higher when compared to the current surveillance protocol, with the highest values for risk-based surveillance protocols. Nonetheless, only S_3V_M0 and S_1V10D_M0 for dog bites had a 95%CI of the BRR that included 1, meaning they were possibly beneficial. For cat bites, only S_1V10D_M0 had a 95%CI of the BRR including 1.

### 3.2. Sensitivity Analyses

**Figure 7:**
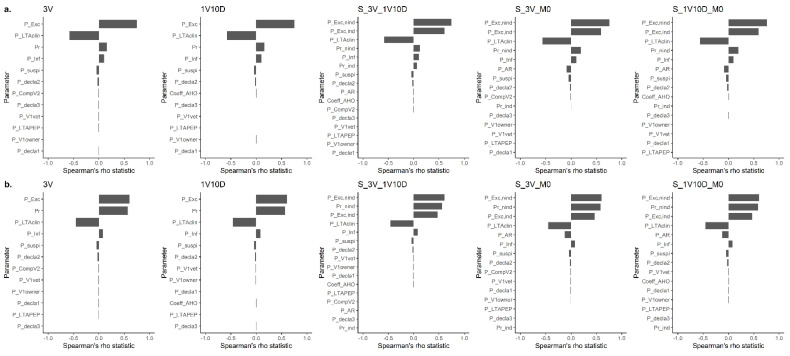
Spearman correlation between the input parameters and the benefit–risk ratio output for dog (**a**) and cat (**b**) bite surveillance protocol models. M0: absence of surveillance protocol; 3V: current French surveillance protocol with three veterinary visits within 15 days; 1V10D: less stringent alternative surveillance protocol relying on one veterinary visit and 10 days of observation at home; and S_3V_1V10D, S_3V_M0, and S_1V10D_M0: risk-based alternative surveillance protocols combining previous protocols (the first protocol of the name is applied to at-risk pets and the second protocol of the name is applied to non-at-risk pets).

The results of the sensitivity analyses based on the Spearman correlation are presented in Figure 7.

For all models, the probability of the biting animal being in a presymptomatic excretion period while incubating rabies ($P_{Exc,nind}$, $P_{Exc,ind}$ and $P_{Exc}$) and the probability of lethal traffic accident when comuting to a veterinary clinic ($P_{LTAclin}$) were parameters whose uncertainty most greatly influenced the uncertainty of the BRR (output of the models). For cat bite surveillance models, the instantaneous prevalences ($Pr_{cat,nind}$, $Pr_{cat,nind},$ and $Pr_{cat}$) also importantly influenced the uncertainty of the BRR.

**Figure 8:**
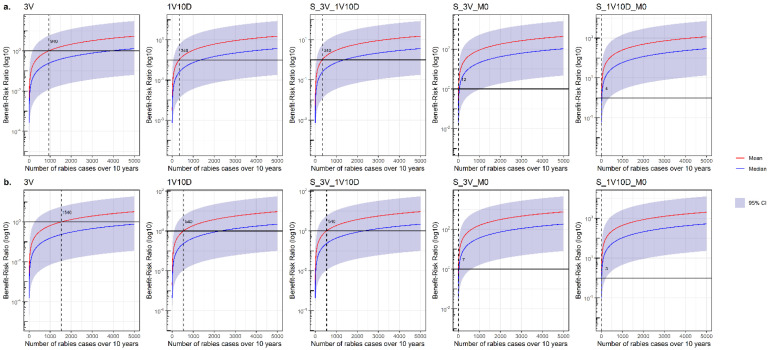
One-way sensitivity analyses on the rabies incidence for dog (**a**) and cat (**b**) bite surveillance protocol models. Dashed lines and associated values indicate the number of rabies cases over 10 years for which the mean of the Benefit–Risk Ratio becomes >1. M0: absence of surveillance protocol; 3V: current French surveillance protocol with three veterinary visits within 15 days; 1V10D: less stringent alternative surveillance protocol relying on one veterinary visit and 10 days of observation at home; and S_3V_1V10D, S_3V_M0, and S_1V10D_M0: risk-based alternative surveillance protocols combining previous protocols (the first protocol of the name is applied to at-risk pets and the second protocol of the name is applied to non-at-risk pets). CI: confidence interval.

One-way sensitivity analyses for rabies incidence are presented in Figure 8. The results highlighted that the mean BRR became >1 for 940 dog rabies cases and 1540 cat rabies cases over 10 years for the current surveillance protocol. This rabies case number value decreased for the less stringent and risk-based surveillance protocols.

An increase in the distance between the owner’s home and the veterinary clinic diminished the BRR for all models, and a decrease in this distance did not allow to have BRR >1 (i.e., beneficial surveillance protocol) for protocols that were not beneficial with the baseline parameterization (Supplementary Material S3). Variations of the owner and veterinarian compliance parameters (probabilities ranging between 0 and 1) had very minor impacts on the BRR (Supplementary Material S3).

## 4. Discussion

We investigated the benefit–risk aspects of the current French surveillance protocol and of alternative surveillance protocols of apparently healthy biting animals for human rabies prevention by a scenario-tree modelling approach. To our knowledge, this is the first assessment of such surveillance protocols, and the results provide useful information to rationalize their implementation modalities. This assessment is strongly rooted in a One Health perspective by evaluating, at the same time, veterinary interventions (veterinary visits for biting animals) and medical interventions (PEP administration); and emphasizes the interest of animal surveillance to appropriately target medical interventions for humans when dealing with zoonoses.

### 4.1. Rationalization of the Surveillance of Apparently Healthy Biting Dogs and Cats for Human Rabies Prevention

The current French surveillance protocol “3V” based on three veterinary visits during a two-week period was shown to be overprotective and even detrimental considering that more deaths were generated (via lethal traffic accidents) than avoided (by prompt PEP administration for people exposed to RABV). Sensitivity analyses showed than an important increase in rabies incidence (≈94 annual dog rabies cases and ≈154 annual cat rabies cases) should occur for this protocol to become beneficial. Such rabies incidence levels could only be observed in the case of an established rabies reservoir (domestic or wildlife with important number of spillover events on dogs and cats). 

Nonetheless, it should be noted that the number of human deaths (linked to rabies and/or traffic accidents) in the context of this surveillance protocol remained extremely low: 15.90 and 18.65 human deaths over 1000 years for the “3V” surveillance protocol for dog bites and cat bites, respectively, almost exclusively due to lethal traffic accidents.

All the alternative surveillance protocols of apparently healthy biting dogs and cats increased the BRR meaning that they were less detrimental than the current protocol “3V”. A reduction of the number of veterinary visits (in “1V10D”) without restricting the surveillance on at-risk animals did not allow a beneficial surveillance protocol to prevent human rabies deaths for the French context. Indeed, only the “S_1V10D_M0” (for both dog and cat bites) and the “S_3V_M0” (for dog bites only) surveillance protocols, which were risk-based, had BRR confidence intervals including 1 (i.e., possibly beneficial). 

It was interesting to note that the “S_3V_M0” surveillance protocol tended to be beneficial since this protocol is currently largely applied by French veterinarians (despite being non-compliant with the legislation) who are prone to conduct their own risk analysis before starting surveillance after a bite [15]. These results highlight the need for, at least, a risk-based surveillance of apparently healthy biting animals in France, as performed in other European countries, such as the UK and Switzerland [21,22], or the possibility to stop this surveillance.

Nonetheless, simply stopping surveillance of biting pets does not seem appropriate for several aspects: such a surveillance protocol (i) raises awareness among pet owners on bite risk and eventually contributes to the identification of dogs with a risk of aggression toward humans since behavior evaluations are required for biting dogs in France [44]; (ii) contributes to reinforcing the link between companion animal veterinarians and veterinary administration (since it deals with the surveillance of a notifiable disease); and (iii) provides the opportunity to communicate with pet owners about zoonotic diseases transmitted by dog and cat bites, including rabies. 

Considering these major aspects, we did not evaluate alternative protocols not requiring veterinary visits (but only phone calls) as implemented in Canada for example [45]. Nonetheless, in terms of rabies risk management only, surveillance protocols without veterinary visits could be more beneficial than those evaluated in this work since they do not require road travels (thus, reducing the probability of lethal traffic accidents).

The at-risk pet selection for risk-based surveillance protocols proposed in our work could eventually be restricted (e.g., targeting only illegal importations and illegal travels for example) in order to further limit the number of observations of biting animals and limit the number of lethal traffic accidents. However, a stricter selection of at-risk pets could increase the probability for a rabies-infected pet to not be targeted by the surveillance protocol.

It is also worth noting that the reduction of the observation period from 15 days (“3V” model) to 10 days (“1V10D” model), as recommended by WHO [23], did not increase the probability of human rabies death in our framework. This could have been expected since long presymptomatic excretion periods are rare, and most are less than three days [24].

The sensitivity analysis results showed that variability on some rabies natural history parameters explained the majority of the uncertainty on the BRR, while compliance parameters, which largely relied on hypotheses, had minor impacts. This indicates that communication reinforcement among pet owners or veterinarians in order to increase compliance would not have an important impact on the benefit–risk aspects of the investigated surveillance protocols of apparently healthy biting animals.

### 4.2. Strengths and Limits of the Study

This modelling approach, despite being a simplification of a more complex reality, allowed us to integrate numerous scenarios as well as numerous parameters with their uncertainty. As a benefit of the surveillance protocols of biting animals, we only considered the direct benefit of the surveillance: avoiding human deaths due to rabies. In any case, indirect benefits could arise, especially if the surveillance includes veterinary visits, as discussed above (e.g., the identification and management of dangerous dogs, communication about zoonoses, and link reinforcement between veterinarians and veterinary administration).

As risks associated with these surveillance protocols, we included lethal traffic accidents when commuting to a veterinary clinic or an anti-rabies center. This approach is unique, as it was only previously incorporated by Ribadeau-Dumas et al. (2015) [19]. Other authors compared the risk-levels of human rabies infection and of lethal traffic accident (without directly including and comparing these outcomes in a framework) to conclude that the probability of rabies infection in Western Europe is very low [18,46]. Comparison of prevented rabies deaths with generated deaths linked to traffic accidents, despite being objective and easy to implement for a benefit–risk assessment, should be interpreted cautiously due to social acceptability concerns. 

In this study, the rabies death risk and traffic accident death risk were put at the same level, whereas societal acceptability is likely much lower for rabies deaths. Indeed, people tend to have lower acceptability for risks from involuntary activities that are not equitable and with fatal consequences (e.g., rabies infection following a bite in this case) whereas risks from voluntary activities that are controllable and not always fatal (e.g., road traffic accidents) are considered as more acceptable [47]. Thus, social acceptability, which is subjective, should be taken into account when changing management measures, which can contribute to increase a poorly socially acceptable risk (even if their implementation reduces a more socially acceptable risk).

It is also important to consider that we did not include in this framework dog/cat scratches, even if there are supposed to be declared and lead to surveillance in France [14]. Indeed, there was no data available for parameterization regarding scratch incidence in the general population and the proportion of scratches declared by pet owners. However, it is possible that including scratches in this framework would have worsened the benefit-risk ratios of the evaluated surveillance protocols. The probability of rabies transmission from scratches is very low and would likely generate additional travel (and thus traffic accidents), even if compliance with the surveillance protocol following a scratch event is likely even lower, for animals presenting an extremely low risk of rabies transmission [48].

The parameterization of the scenario-tree models involved numerous data sources, some of which could be prone to bias. Parameters relying on a survey (i.e., with a sampling process) could particularly produce biased estimates due to non-representativeness of the used sample (sampling bias). This could be the case for the online survey results used here. However, a post-stratification process was used to appropriately weight observations and limit the impact of this convenience sample (i.e., non-probability sampling method) on the quality of the estimators [40,49,50,51,52]. Moreover, sensitivity analyses showed that theses parameters had minor impacts on the uncertainty of the output.

The parameters regarding the natural history of rabies have often relied on few and sometimes old studies. This was especially the case for the presymptomatic excretion period, which also conditioned the probability for a pet incubating rabies to be in presymptomatic excretion [24,32,33]. However, the biological variability identified through available data was included in the definition of the distributions, and the impact of such variability was assessed through the sensitivity analyses (based on the Spearman correlation).

For parameters that included a traffic accident probability, we assumed that all travels to veterinary clinics or anti-rabies centers were performed using cars or road public transportation since there was no data available for parameterization of the death probability for displacements on foot or via public rail transportation. This seems to be a reasonable assumption since only very short travels would be made on foot and the use of cars may be favored for the transportation of pets (for travels to veterinary clinics). Moreover, we used a mean value for the lethal traffic accident probability, while, in reality, this probability is likely dependent on several variables (the road condition, geographical area, time of travel, etc.)

When possible, we distinguished between dogs and cats for parameterization but this was not always feasible due to the scarcity of data about cats. For example, there was no data available for anatomic sites of cat bites in France [35]. For the rabies natural history parameters, we chose to combine dog and cat data due to scarcity and because no significant difference in parameters between the two species was identified (e.g., the incubation period [31]).

## 5. Conclusions and Perspectives

Through a scenario-tree modelling approach, we showed that the current regulatory surveillance protocol of apparently healthy biting cats/dogs for preventing human rabies infections in France was no longer justifiable. Indeed, this surveillance protocol could actually be generating more human deaths due to traffic accidents occurring during travels imposed by the protocol rather than avoiding human rabies death by guiding human bite victims to anti-rabies centers. Such results could only be observed because the risk of rabies is extremely low in France (lower than the death probability associated with traffic accidents). 

In areas where rabies is enzootic, especially with a dog reservoir, an observation period for biting dogs/cats could prove beneficial (as highlighted by our sensitivity analyses) and should be considered, as its implementation could contribute to a decrease in human deaths from rabies. We showed that the surveillance of biting animals in France should be risk-based, specifically with regard to targeting cats/dogs with a history of trips outside of French territory. 

Such data could help policy-makers in updating the legislation regarding dog/cat bite management by establishing measures in line with the rabies epidemiological context, based on tangible scientific elements. This benefit–risk assessment was the first step to evaluate the surveillance protocol of apparently healthy biting animals in France. Other less-stringent surveillance protocols could be investigated and compared with this method, and the models proposed here could be extended with a cost-effectiveness evaluation for beneficial surveillance protocols in order to include economic aspects.

## Figures and Tables

**Table 1:** Risk-based alternative surveillance protocols.

Name of the Alternative Surveillance Protocol	Surveillance Protocol for At-Risk Pets	Surveillance Protocol for Non-At-Risk Pets
S_3V_1V10D	Current surveillance protocol with three veterinary visits (3V)	Less stringent alternative surveillance protocol with one veterinary visit and 10 days of observation (1V10D)
S_3V_M0	Current surveillance protocol with three veterinary visits (3V)	None (M0)
S_1V10D_M0	Less stringent alternative surveillance protocol with one veterinary visit and 10 days of observation (1V10D)	None (M0)

**Table 2:** Parameters to define the at-risk French pet population.

Parameter	Species	Name	Value	Data Source/Reference
Probability for a pet to have travelled outside the EU in the previous year	dog	$P_{travnEU,dog}$	Beta (5.3 + 1; 1947.4 + 1)	[39]
	cat	$P_{travnEU,cat}$	Beta (6.3 + 1; 2944.6 + 1)	[39]
Probability for a pet to have travelled illegally in the EU in the previous year	dog	$P_{travillEU,dog}$	Beta (6.2 + 1; 1936.6 + 1)	[39]
	cat	$P_{travillEU,cat}$	Beta (3.2 + 1; 2947.4 + 1)	[39]
Probability for a pet to have been imported in the previous year	dog	$P_{imp,dog}$	Beta (25,818 + 1; $POP_{dog}$ −25,818 + 1)	I-CAD and [7]
	cat	$P_{imp,cat}$	Beta (5706 + 1; $POP_{cat}$ −5706 + 1)	I-CAD and [7]

**Table 3:** Compliance parameters and their distributions.

Parameter	Name	Distribution	Reference/Rationale
Probability of starting a surveillance after a bite (i.e., attend a first veterinary visit)	$P_{compV1,dog}$ $P_{compV1,cat}$	$P_{compV1}=P_{V1owner}\times P_{V1vet}$ With $P_{V1owner}$ as the probability that an owner declares a bite to a veterinarian and $P_{V1vet}$ as the probability that the veterinarian starts a surveillance (with a first clinical examination)	Survey (Supplementary Material S1) and non-parametric distributions according to [15]
		For dogs: $P_{V1owner}=$ Beta (16.9 + 1; 48.2 + 1) and $P_{V1vet}=\mathrm{PERT}\left(0.08;\;0.70;\;1\right)$	
		For cats: $P_{V1owner}=$ Beta (31.3 + 1; 140.0 + 1) and $P_{V1vet}=\mathrm{PERT}\left(0,\;0.15;\;1\right)$	
Probability of an owner attending the second veterinary visit (V2) in the protocol “3V”	$P_{compV2}$	$P_{compV2}\times P_{compV3}=\mathrm{PERT}\left(0.8;0.94;0.99\right)$ and $P_{compV3}=P_{compV2\;}+\frac{2}{3}\left(1-P_{compV2}\right)$	Non-parametric distribution according to [15] and authors’ assumption of a better compliance for V3 (2/3 gain over the compliance defect $\left(1-P_{compV2}\right)$ of V2)
Probability of an owner attending the third veterinary visit (V3) in the protocol “3V”	$P_{compV3}$		
Probability of an owner declaring clinical signs after completing a surveillance	$P_{decla1}$	PERT (0.9; 1; 1)	Authors’ assumption (owners were aware of rabies risk, so we made the hypothesis of very good compliance)
Probability of an owner declaring clinical signs after early termination of a surveillance	$P_{decla2}$	PERT (0.7; 0.8; 1)	Authors’ assumption (owners were aware of rabies risk, so we made the hypothesis of good compliance)
Probability of an owner declaring clinical signs if no surveillance has been started	$P_{decla3}$	PERT (0.6; 0.8; 1)	Authors’ assumption (high probability since owners are likely to seek care if neurological signs occur)
Probability of a veterinarian suspecting rabies based on clinical signs and declaring a suspicion to the administration	$P_{suspi}$	PERT (0.05; 0.25; 0.5)	Authors’ assumption (low probability since rabies clinical signs are not specific and the rabies probability of occurrence in France is very low)
Probability of an owner declaring clinical signs during an at-home observation period in the protocol “1V10D”	$P_{comp10D}$	$P_{comp10D}=P_{decla1}\times Coeff_{AHO}$ with $Coeff_{AHO}=\;\mathrm{PERT}\left(0.8;0.95;\;1\right)$	Authors’ assumption (probability lower than $P_{declal1}$ since the protocol “1V10D” was less strict and gave less opportunities to present to the owner the importance of the surveillance)

## Data Availability

No new data were created or analyzed in this study. Data sharing is not applicable to this article.

## Supplementary Materials

The following are available online at https://www.mdpi.com/article/10.3390/vetsci8070132/s1, Supplementary Material S1: Survey on dog and cat bites. Supplementary Material S2: Probabilities associated with scenario-tree branches and mortality probabilities. Supplementary Material S3: Results of the sensitivity analyses.
