# Supplementary material for: Benefit–Risk Assessment of the French Surveillance Protocol of Apparently Healthy Biting Dogs and Cats for Human Rabies Prevention
Source: Vet Sci. 2021 Jul 13;8(7):132. doi: 10.3390/vetsci8070132 (PMC8309990; doi:10.3390/vetsci8070132)
Supplement: Supplementary file 1 [file vetsci-08-00132-s001.zip › vetsci-1254204-supplementary.pdf]

Supplementary Materials - Benefit–Risk Assessment of the French Surveillance Protocol of Apparently Healthy Biting Dogs and Cats for Human Rabies Prevention

Supplementary Material A. Survey on dog and cat bites

In order to obtain parameter values about dog and cat bites, we used results from a survey conducted among the general French population. Results about pet owner practices from this survey are presented elsewhere [1] and are also used in this work (for the probability for a pet to be "at-risk" for rabies). We describe here the materials and methods and present the main results about dog and cat bites.

Materials and methods

The survey targeted people living in France mainland over 18 years of age. An online questionnaire which used Sphinx™ platform was designed to collect data about pet management practices and pet travels [1], but also dog and cat bites on humans. Only bites with skin-punctures were considered.

Observations were appropriately weighted following a post-stratification process using three variables (also called raking) as described by Lumley (2011) [2]. The three variables used for the raking process were the socio-professional category-age variable, pet ownership variable, gender variable, in order to reflect the structure of the French population (as presented in Tables A1, A2, and A3). People working in a veterinary profession were considered as a separate stratum (representing 86,875 people), given their specific risk for dog and cat bites, and were not considered in the raking process but accounted for in the post-stratification (i.e. weighting).

Table A1. Structure of the French population for age and socio-professional category [3]

Age	Socio-professional category	% among French people
15-29	Lower socio-professional category	8.05%
15-29	Higher socio-professional category	1.43%
15-29	Student	7.98%
15-29	No activity	3.75%
30-39	Lower socio-professional category	9.09%
30-39	Higher socio-professional category	3.15%
30-39	No activity (including students and retired)	3.00%
40-49	Lower socio-professional category	9.81%
40-49	Higher socio-professional category	3.60%
40-49	No activity (including students and retired)	2.71%
50-59	Lower socio-professional category	9.20%
50-59	Higher socio-professional category	3.27%
50-59	No activity (including students and retired)	3.85%
≥60	Lower socio-professional category	1.85%
≥60	Higher socio-professional category	1.16%
≥60	No activity (including students and retired)	27.94%

Table A2. Structure of the French population for gender [4]

Gender	% among French people

Man	47.70%
Woman	52.30%

Table A3. Structure of the French population for pet ownership [5]

Pet ownership	% among French people
Owns a dog and/or a cat	42.20%
Does not own a dog nor a cat	57.80%

Descriptive analyses were performed on post-stratified observations. Results were presented in the form of proportions or means with their 95% confidence intervals (95%CI), with variance estimates taking into account the post-stratification process as described by Valliant (1993) [6].

Results on dog and cat bites

Participation to the study

2,384 questionnaires were completed and 2,336 met the inclusion criteria (34 concerned respondents outside mainland France, 14 concerned respondents that did not meet the age criterion).

Results about dog and cat bite incidence

Among the participants (and after the post-stratification process), 3.1% [2.3; 4.1]$_{95\%CI}$ had been bitten at least once by a dog in the five previous years and 7.8% [6.3; 9.6]$_{95\%CI}$ had been bitten at least once by a cat in the five previous years. Bite incidence results are presented in Table A4.

Table A4. Dog and cat bite annual incidences

Species	Mean annual bite number per person	Standard deviation	95% Confidence interval
Dog	1.04×10^{-2}	1.10×10^{-1}	[5.29×10^{-3}; 1.55×10^{-2}]
Cat	4.30×10^{-2}	2.47×10^{-1}	[3.19×10^{-2}; 5.41×10^{-2}]

Among people bitten at least once in the five previous years, 25.9% [13.2; 42.6]$_{95\%CI}$ and 18.2% [11.5; 26.9]$_{95\%CI}$ declared that at least one biting dog and at least one biting cat, respectively, was presented to a veterinarian.

References

1. Crozet, G.; Lacoste, M.-L.; Rivière, J.; Robardet, E.; Cliquet, F.; Dufour, B. Management Practices of Dog and Cat Owners in France (Pet Traveling, Animal Contact Rates and Medical Monitoring): Impacts on the Introduction and the Spread of Directly Transmitted Infectious Pet Diseases. *Transbound Emerg Dis* **2021**, doi:10.1111/tbed.14088.
2. Lumley, T. *Complex Surveys: A Guide to Analysis Using R*; John Wiley & Sons, 2011; ISBN 978-1-118-21093-2.
3. INSEE. Activité, Emploi et Chômage En 2018. Available online: https://www.insee.fr/fr/statistiques/4191029#consulter (accessed on 13 April 2020).
4. INSEE. Pyramide Des Âges 2018. Available online: https://www.insee.fr/fr/statistiques/2381472 (accessed on 13 April 2020).
5. FACCO. Les Chiffres de La Possession Animale En France. Available online: https://www.facco.fr/les-chiffres/.
6. Valliant, R. Poststratification and Conditional Variance Estimation. *Journal of the American Statistical Association* **1993**, *88*, 89–96, doi:10.2307/2290701.

Supplementary Material B. Probabilities associated with scenarios-tree branches and mortality probabilities

Model "M0"

Probabilities associated with branches (n=7) of the scenario-tree model

$M0_1 = Pr \times P_{Exc} \times P_{inf} \times P_{CSexc,15} \times ((1 - P_{decla3}) + P_{decla3} \times (1 - P_{LTAclin}) \times (1 - P_{suspi}))$
$M0_2 = Pr \times P_{Exc} \times P_{inf} \times P_{CSexc,15} \times P_{decla3} \times P_{LTAclin}$
$M0_3 = Pr \times P_{Exc} \times P_{inf} \times P_{CSexc,15} \times P_{decla3} \times (1 - P_{LTAclin}) \times P_{suspi} \times P_{LTAPEP}$
$M0_4 = Pr \times P_{Exc} \times (1 - P_{inf}) \times P_{CSexc,15} \times P_{decla3} \times P_{LTAclin}$
$M0_5 = Pr \times P_{Exc} \times (1 - P_{inf}) \times P_{CSexc,15} \times P_{decla3} \times (1 - P_{LTAclin}) \times P_{suspi} \times P_{LTAPEP}$
$M0_6 = Pr \times (1 - P_{Exc}) \times P_{inf} \times P_{CSnexc,15} \times P_{decla3} \times P_{LTAclin}$
$M0_7 = Pr \times (1 - P_{Exc}) \times P_{inf} \times P_{CSnexc,15} \times P_{decla3} \times (1 - P_{LTAclin}) \times P_{suspi} \times P_{LTAPEP}$

Human death probabilities

$P_{M0rabies} = M0_1$ (Rabies death probability)
$P_{M0LTA} = \sum_{i \in \{2:7\}} M0_i$ (Traffic accident death probability)

Model "3V"

Probabilities associated with branches (n=42) of the scenario-tree model

$M3V_1 = Pr \times P_{Exc} \times P_{inf} \times P_{compV1} \times (1 - P_{LTAclin}) \times (1 - P_{CSexc,1}) \times P_{compV2} \times (1 - P_{LTAclin}) \times (1 - P_{CSexc,7}) \times P_{compV3} \times (1 - P_{LTAclin}) \times P_{CSexc,15} \times P_{LTAPEP}$
$M3V_2 = Pr \times P_{Exc} \times P_{inf} \times P_{compV1} \times (1 - P_{LTAclin}) \times (1 - P_{CSexc,1}) \times P_{compV2} \times (1 - P_{LTAclin}) \times (1 - P_{CSexc,7}) \times P_{compV3} \times P_{LTAclin}$
$M3V_3 = Pr \times P_{Exc} \times P_{inf} \times P_{compV1} \times (1 - P_{LTAclin}) \times (1 - P_{CSexc,1}) \times P_{compV2} \times (1 - P_{LTAclin}) \times (1 - P_{CSexc,7}) \times (1 - P_{compV3}) \times P_{CSexc,15} \times P_{decla2} \times (1 - P_{LTAclin}) \times P_{LTAPEP}$
$M3V_4 = Pr \times P_{Exc} \times P_{inf} \times P_{compV1} \times (1 - P_{LTAclin}) \times (1 - P_{CSexc,1}) \times P_{compV2} \times (1 - P_{LTAclin}) \times (1 - P_{CSexc,7}) \times (1 - P_{compV3}) \times P_{CSexc,15} \times P_{decla2} \times P_{LTAclin}$
$M3V_5 = Pr \times P_{Exc} \times P_{inf} \times P_{compV1} \times (1 - P_{LTAclin}) \times (1 - P_{CSexc,1}) \times P_{compV2} \times (1 - P_{LTAclin}) \times (1 - P_{CSexc,7}) \times (1 - P_{compV3}) \times P_{CSexc,15} \times (1 - P_{decla2})$
$M3V_6 = Pr \times P_{Exc} \times P_{inf} \times P_{compV1} \times (1 - P_{LTAclin}) \times (1 - P_{CSexc,1}) \times P_{compV2} \times (1 - P_{LTAclin}) \times P_{CSexc,7} \times P_{LTAPEP}$
$M3V_7 = Pr \times P_{Exc} \times P_{inf} \times P_{compV1} \times (1 - P_{LTAclin}) \times (1 - P_{CSexc,1}) \times P_{compV2} \times P_{LTAclin}$
$M3V_8 = Pr \times P_{Exc} \times P_{inf} \times P_{compV1} \times (1 - P_{LTAclin}) \times (1 - P_{CSexc,1}) \times (1 - P_{compV2}) \times P_{CSexc,15} \times P_{decla2} \times (1 - P_{LTAclin}) \times P_{LTAPEP}$
$M3V_9 = Pr \times P_{Exc} \times P_{inf} \times P_{compV1} \times (1 - P_{LTAclin}) \times (1 - P_{CSexc,1}) \times (1 - P_{compV2}) \times P_{CSexc,15} \times P_{decla2} \times P_{LTAclin}$
$M3V_{10} = Pr \times P_{Exc} \times P_{inf} \times P_{compV1} \times (1 - P_{LTAclin}) \times (1 - P_{CSexc,1}) \times (1 - P_{compV2}) \times P_{CSexc,15} \times (1 - P_{decla2})$
$M3V_{11} = Pr \times P_{Exc} \times P_{inf} \times P_{compV1} \times (1 - P_{LTAclin}) \times P_{CSexc,1} \times P_{LTAPEP}$
$M3V_{12} = Pr \times P_{Exc} \times P_{inf} \times P_{compV1} \times P_{LTAclin}$
$M3V_{13} = Pr \times P_{Exc} \times P_{inf} \times ((1 - P_{V1owner}) + P_{V1owner} \times (1 - P_{V1vet})) \times P_{CSexc,15} \times P_{decla3} \times (1 - P_{LTAclin}) \times P_{suspi} \times P_{LTAPEP}$
$M3V_{14} = Pr \times P_{Exc} \times P_{inf} \times ((1 - P_{V1owner}) + P_{V1owner} \times (1 - P_{V1vet})) \times P_{CSexc,15} \times P_{decla3} \times P_{LTAclin}$
$M3V_{15} = Pr \times P_{Exc} \times P_{inf} \times ((1 - P_{V1owner}) + P_{V1owner} \times (1 - P_{V1vet})) \times P_{CSexc,15} \times ((1 - P_{decla3}) + P_{decla3} \times (1 - P_{LTAclin}) \times (1 - P_{suspi}))$
$M3V_{16} = Pr \times P_{Exc} \times (1 - P_{inf}) \times P_{compV1} \times (1 - P_{LTAclin}) \times (1 - P_{CSexc,1}) \times P_{compV2} \times (1 - P_{LTAclin}) \times (1 - P_{CSexc,7}) \times P_{compV3} \times (1 - P_{LTAclin}) \times P_{CSexc,15} \times P_{LTAPEP}$
$M3V_{17} = Pr \times P_{Exc} \times (1 - P_{inf}) \times P_{compV1} \times (1 - P_{LTAclin}) \times (1 - P_{CSexc,1}) \times P_{compV2} \times (1 - P_{LTAclin}) \times (1 - P_{CSexc,7}) \times P_{compV3} \times P_{LTAclin}$

$$M3V_{18} = Pr \times P_{Exc} \times (1-P_{inf}) \times P_{compV1} \times (1-P_{LTAclin}) \times \left(1-P_{CSexc,1}\right) \times P_{compV2} \times (1-P_{LTAclin}) \times \left(1-P_{CSexc,7}\right) \times (1-P_{compV3}) \times P_{CSexc,15} \times P_{decla2} \times (1-P_{LTAclin}) \times P_{LTAPEP}$$
$$M3V_{19} = Pr \times P_{Exc} \times (1-P_{inf}) \times P_{compV1} \times (1-P_{LTAclin}) \times \left(1-P_{CSexc,1}\right) \times P_{compV2} \times (1-P_{LTAclin}) \times \left(1-P_{CSexc,7}\right) \times (1-P_{compV3}) \times P_{CSexc,15} \times P_{decla2} \times P_{LTAclin}$$
$$M3V_{20} = Pr \times P_{Exc} \times (1-P_{inf}) \times P_{compV1} \times (1-P_{LTAclin}) \times \left(1-P_{CSexc,1}\right) \times P_{compV2} \times (1-P_{LTAclin}) \times P_{CSexc,7} \times P_{LTAPEP}$$
$$M3V_{21} = Pr \times P_{Exc} \times (1-P_{inf}) \times P_{compV1} \times (1-P_{LTAclin}) \times \left(1-P_{CSexc,1}\right) \times P_{compV2} \times P_{LTAclin}$$
$$M3V_{22} = Pr \times P_{Exc} \times (1-P_{inf}) \times P_{compV1} \times (1-P_{LTAclin}) \times \left(1-P_{CSexc,1}\right) \times (1-P_{compV2}) \times P_{CSexc,15} \times P_{decla2} \times (1-P_{LTAclin}) \times P_{LTAPEP}$$
$$M3V_{23} = Pr \times P_{Exc} \times (1-P_{inf}) \times P_{compV1} \times (1-P_{LTAclin}) \times \left(1-P_{CSexc,1}\right) \times (1-P_{compV2}) \times P_{CSexc,15} \times P_{decla2} \times P_{LTAclin}$$
$$M3V_{24} = Pr \times P_{Exc} \times (1-P_{inf}) \times P_{compV1} \times (1-P_{LTAclin}) \times P_{CSexc,1} \times P_{LTAPEP}$$
$$M3V_{25} = Pr \times P_{Exc} \times (1-P_{inf}) \times P_{compV1} \times P_{LTAclin}$$
$$M3V_{26} = Pr \times P_{Exc} \times (1-P_{inf}) \times ((1-P_{V1owner}) + P_{V1owner} \times (1-P_{V1vet})) \times P_{CSexc,15} \times P_{decla3} \times (1-P_{LTAclin}) \times P_{suspi} \times P_{LTAPEP}$$
$$M3V_{27} = Pr \times P_{Exc} \times (1-P_{inf}) \times ((1-P_{V1owner}) + P_{V1owner} \times (1-P_{V1vet})) \times P_{CSexc,15} \times P_{decla3} \times P_{LTAclin}$$
$$M3V_{28} = Pr \times (1-P_{Exc}) \times P_{compV1} \times (1-P_{LTAclin}) \times \left(1-P_{CSnexc,1}\right) \times P_{compV2} \times (1-P_{LTAclin}) \times \left(1-P_{CSnexc,7}\right) \times P_{compV3} \times (1-P_{LTAclin}) \times P_{CSnexc,15} \times P_{LTAPEP}$$
$$M3V_{29} = Pr \times (1-P_{Exc}) \times P_{compV1} \times (1-P_{LTAclin}) \times \left(1-P_{CSnexc,1}\right) \times P_{compV2} \times (1-P_{LTAclin}) \times \left(1-P_{CSnexc,7}\right) \times P_{compV3} \times P_{LTAclin}$$
$$M3V_{30} = Pr \times (1-P_{Exc}) \times P_{compV1} \times (1-P_{LTAclin}) \times \left(1-P_{CSnexc,1}\right) \times P_{compV2} \times (1-P_{LTAclin}) \times \left(1-P_{CSnexc,7}\right) \times (1-P_{compV3}) \times P_{CSnexc,15} \times P_{decla2} \times (1-P_{LTAclin}) \times P_{LTAPEP}$$
$$M3V_{31} = Pr \times (1-P_{Exc}) \times P_{compV1} \times (1-P_{LTAclin}) \times \left(1-P_{CSnexc,1}\right) \times P_{compV2} \times (1-P_{LTAclin}) \times \left(1-P_{CSnexc,7}\right) \times (1-P_{compV3}) \times P_{CSnexc,15} \times P_{decla2} \times P_{LTAclin}$$
$$M3V_{32} = Pr \times (1-P_{Exc}) \times P_{compV1} \times (1-P_{LTAclin}) \times \left(1-P_{CSnexc,1}\right) \times P_{compV2} \times (1-P_{LTAclin}) \times P_{CSnexc,7} \times P_{LTAPEP}$$
$$M3V_{33} = Pr \times (1-P_{Exc}) \times P_{compV1} \times (1-P_{LTAclin}) \times \left(1-P_{CSnexc,1}\right) P_{compV2} \times P_{LTAclin}$$
$$M3V_{34} = Pr \times (1-P_{Exc}) \times P_{compV1} \times (1-P_{LTAclin}) \times \left(1-P_{CSnexc,1}\right) \times (1-P_{compV2}) \times P_{CSnexc,15} \times P_{decla2} \times (1-P_{LTAclin}) \times P_{LTAPEP}$$
$$M3V_{35} = Pr \times (1-P_{Exc}) \times P_{compV1} \times (1-P_{LTAclin}) \times \left(1-P_{CSnexc,1}\right) \times (1-P_{compV2}) \times P_{CSnexc,15} \times P_{decla2} \times P_{LTAclin}$$
$$M3V_{36} = Pr \times (1-P_{Exc}) \times P_{compV1} \times (1-P_{LTAclin}) \times P_{CSnexc,1} \times P_{LTAPEP}$$
$$M3V_{37} = Pr \times (1-P_{Exc}) \times P_{compV1} \times P_{LTAclin}$$
$$M3V_{38} = Pr \times (1-P_{Exc}) \times ((1-P_{V1owner}) + P_{V1owner} \times (1-P_{V1vet})) \times P_{CSnexc,15} \times P_{decla3} \times (1-P_{LTAclin}) \times P_{suspi} \times P_{LTAPEP}$$
$$M3V_{39} = Pr \times (1-P_{Exc}) \times ((1-P_{V1owner}) + P_{V1owner} \times (1-P_{V1vet})) \times P_{CSnexc,15} \times P_{decla3} \times P_{LTAclin}$$
$$M3V_{40} = (1-Pr) \times P_{compV1} \times (1-P_{LTAclin}) \times P_{compV2} \times (1-P_{LTAclin}) \times P_{compV3} \times P_{LTAclin}$$
$$M3V_{41} = (1-Pr) \times P_{compV1} \times (1-P_{LTAclin}) \times P_{compV2} \times P_{LTAclin}$$
$$M3V_{42} = (1-Pr) \times P_{compV1} \times P_{LTAclin}$$

Human death probabilities

$P_{M3Vrabies} = M3V_5 + M3V_{10} + M3V_{15}$ (Rabies death probability)

$P_{M3VLTA} = \sum_{i \in \{1:42\} \setminus \{5,10,15\}} M3V_i$ (Traffic accident death probability)

Model "1V10D"

Probabilities associated with branches (n=31) of the scenario-tree model

$$M1V10D_1 = Pr \times P_{Exc} \times P_{inf} \times P_{compV1} \times (1-P_{LTAclin}) \times P_{CSexc,1} \times P_{LTAPEP}$$
$$M1V10D_2 = Pr \times P_{Exc} \times P_{inf} \times P_{compV1} \times (1-P_{LTAclin}) \times \left(1-P_{CSexc,1}\right) \times P_{comp10D} \times P_{CSexc,10} \times P_{LTAPEP}$$

$$M1V10D_3 = Pr \times P_{Exc} \times P_{inf} \times P_{compV1} \times (1 - P_{LTAclin}) \times \left(1 - P_{CSexc,1}\right) \times P_{comp10D} \times (1 - P_{CSexc,10}) \times P_{CSexc,15} \times P_{decla1} \times (1 - P_{LTAclin}) \times P_{LTAPEP}$$
$$M1V10D_4 = Pr \times P_{Exc} \times P_{inf} \times P_{compV1} \times (1 - P_{LTAclin}) \times \left(1 - P_{CSexc,1}\right) \times P_{comp10D} \times (1 - P_{CSexc,10}) \times P_{CSexc,15} \times P_{decla1} \times P_{LTAclin}$$
$$M1V10D_5 = Pr \times P_{Exc} \times P_{inf} \times P_{compV1} \times (1 - P_{LTAclin}) \times \left(1 - P_{CSexc,1}\right) \times P_{comp10D} \times (1 - P_{CSexc,10}) \times P_{CSexc,15} \times (1 - P_{decla1})$$
$$M1V10D_6 = Pr \times P_{Exc} \times P_{inf} \times P_{compV1} \times (1 - P_{LTAclin}) \times \left(1 - P_{CSexc,1}\right) \times (1 - P_{comp10D}) \times P_{CSexc,15} \times P_{decla2} \times (1 - P_{LTAclin}) \times P_{LTAPEP}$$
$$M1V10D_7 = Pr \times P_{Exc} \times P_{inf} \times P_{compV1} \times (1 - P_{LTAclin}) \times \left(1 - P_{CSexc,1}\right) \times (1 - P_{comp10D}) \times P_{CSexc,15} \times P_{decla2} \times P_{LTAclin}$$
$$M1V10D_8 = Pr \times P_{Exc} \times P_{inf} \times P_{compV1} \times (1 - P_{LTAclin}) \times \left(1 - P_{CSexc,1}\right) \times (1 - P_{comp10D}) \times P_{CSexc,15} \times (1 - P_{decla2})$$
$$M1V10D_9 = Pr \times P_{Exc} \times P_{inf} \times P_{compV1} \times P_{LTAclin}$$
$$M1V10D_{10} = Pr \times P_{Exc} \times P_{inf} \times ((1 - P_{V1owner}) + P_{V1owner} \times (1 - P_{V1vet})) \times P_{CSexc,15} \times P_{decla3} \times (1 - P_{LTAclin}) \times P_{suspi} \times P_{LTAPEP}$$
$$M1V10D_{11} = Pr \times P_{Exc} \times P_{inf} \times ((1 - P_{V1owner}) + P_{V1owner} \times (1 - P_{V1vet})) \times P_{CSexc,15} \times P_{decla3} \times P_{LTAclin}$$
$$M1V10D_{12} = Pr \times P_{Exc} \times P_{inf} \times ((1 - P_{V1owner}) + P_{V1owner} \times (1 - P_{V1vet})) \times P_{CSexc,15} \times ((1 - P_{decla3}) + P_{decla3} \times (1 - P_{LTAclin}) \times \left(1 - P_{suspi}\right))$$
$$M1V10D_{13} = Pr \times P_{Exc} \times (1 - P_{inf}) \times P_{compV1} \times (1 - P_{LTAclin}) \times P_{CSexc,1} \times P_{LTAPEP}$$
$$M1V10D_{14} = Pr \times P_{Exc} \times (1 - P_{inf}) \times P_{compV1} \times (1 - P_{LTAclin}) \times \left(1 - P_{CSexc,1}\right) \times P_{comp10D} \times P_{CSexc,10} \times P_{LTAPEP}$$
$$M1V10D_{15} = Pr \times P_{Exc} \times (1 - P_{inf}) \times P_{compV1} \times (1 - P_{LTAclin}) \times \left(1 - P_{CSexc,1}\right) \times P_{comp10D} \times (1 - P_{CSexc,10}) \times P_{CSexc,15} \times P_{decla1} \times (1 - P_{LTAclin}) \times P_{LTAPEP}$$
$$M1V10D_{16} = Pr \times P_{Exc} \times (1 - P_{inf}) \times P_{compV1} \times (1 - P_{LTAclin}) \times \left(1 - P_{CSexc,1}\right) \times P_{comp10D} \times (1 - P_{CSexc,10}) \times P_{CSexc,15} \times P_{decla1} \times P_{LTAclin}$$
$$M1V10D_{17} = Pr \times P_{Exc} \times (1 - P_{inf}) \times P_{compV1} \times (1 - P_{LTAclin}) \times \left(1 - P_{CSexc,1}\right) \times (1 - P_{comp10D}) \times P_{CSexc,15} \times P_{decla2} \times (1 - P_{LTAclin}) \times P_{LTAPEP}$$
$$M1V10D_{18} = Pr \times P_{Exc} \times (1 - P_{inf}) \times P_{compV1} \times (1 - P_{LTAclin}) \times \left(1 - P_{CSexc,1}\right) \times (1 - P_{comp10D}) \times P_{CSexc,15} \times P_{decla2} \times P_{LTAclin}$$
$$M1V10D_{19} = Pr \times P_{Exc} \times (1 - P_{inf}) \times P_{compV1} \times P_{LTAclin}$$
$$M1V10D_{20} = Pr \times P_{Exc} \times (1 - P_{inf}) \times ((1 - P_{V1owner}) + P_{V1owner} \times (1 - P_{V1vet})) \times P_{CSexc,15} \times P_{decla3} \times (1 - P_{LTAclin}) \times P_{suspi} \times P_{LTAPEP}$$
$$M1V10D_{21} = Pr \times P_{Exc} \times (1 - P_{inf}) \times ((1 - P_{V1owner}) + P_{V1owner} \times (1 - P_{V1vet})) \times P_{CSexc,15} \times P_{decla3} \times P_{LTAclin}$$
$$M1V10D_{22} = Pr \times (1 - P_{Exc}) \times P_{compV1} \times (1 - P_{LTAclin}) \times P_{CSnexc,1} \times P_{LTAPEP}$$
$$M1V10D_{23} = Pr \times (1 - P_{Exc}) \times P_{compV1} \times (1 - P_{LTAclin}) \times \left(1 - P_{CSnexc,1}\right) \times P_{comp10D} \times P_{CSnexc,10} \times P_{LTAPEP}$$
$$M1V10D_{24} = Pr \times (1 - P_{Exc}) \times P_{compV1} \times (1 - P_{LTAclin}) \times \left(1 - P_{CSnexc,1}\right) \times P_{comp10D} \times (1 - P_{CSnexc,10}) \times P_{CSnexc,15} \times P_{decla1} \times (1 - P_{LTAclin}) \times P_{LTAPEP}$$
$$M1V10D_{25} = Pr \times (1 - P_{Exc}) \times P_{compV1} \times (1 - P_{LTAclin}) \times \left(1 - P_{CSnexc,1}\right) \times P_{comp10D} \times (1 - P_{CSnexc,10}) \times P_{CSnexc,15} \times P_{decla1} \times P_{LTAclin}$$
$$M1V10D_{26} = Pr \times (1 - P_{Exc}) \times P_{compV1} \times (1 - P_{LTAclin}) \times \left(1 - P_{CSnexc,1}\right) \times (1 - P_{comp10D}) \times P_{CSnexc,15} \times P_{decla2} \times (1 - P_{LTAclin}) \times P_{LTAPEP}$$
$$M1V10D_{27} = Pr \times (1 - P_{Exc}) \times P_{compV1} \times (1 - P_{LTAclin}) \times \left(1 - P_{CSnexc,1}\right) \times (1 - P_{comp10D}) \times P_{CSnexc,15} \times P_{decla2} \times P_{LTAclin}$$
$$M1V10D_{28} = Pr \times (1 - P_{Exc}) \times P_{compV1} \times P_{LTAclin}$$
$$M1V10D_{29} = Pr \times (1 - P_{Exc}) \times ((1 - P_{V1owner}) + P_{V1owner} \times (1 - P_{V1vet})) \times P_{CSnexc,15} \times P_{decla3} \times (1 - P_{LTAclin}) \times P_{suspi} \times P_{LTAPEP}$$
$$M1V10D_{30} = Pr \times (1 - P_{Exc}) \times ((1 - P_{V1owner}) + P_{V1owner} \times (1 - P_{V1vet})) \times P_{CSnexc,15} \times P_{decla3} \times P_{LTAclin}$$
$$M1V10D_{31} = (1 - Pr) \times P_{compV1} \times P_{LTAclin}$$

Human death probabilities

$$P_{M1V10Drabies} = M1V10D_5 + M1V10D_8 + M1V10D_{12}$$ (Rabies death probability)

$$P_{M1V10DLTA} = \sum_{i\,\in\{1:31\}\setminus\{5,8,12\}} M1V10D_i \qquad \text{(Traffic accident death probability)}$$

Supplementary Material C. Results of the sensitivity analyses

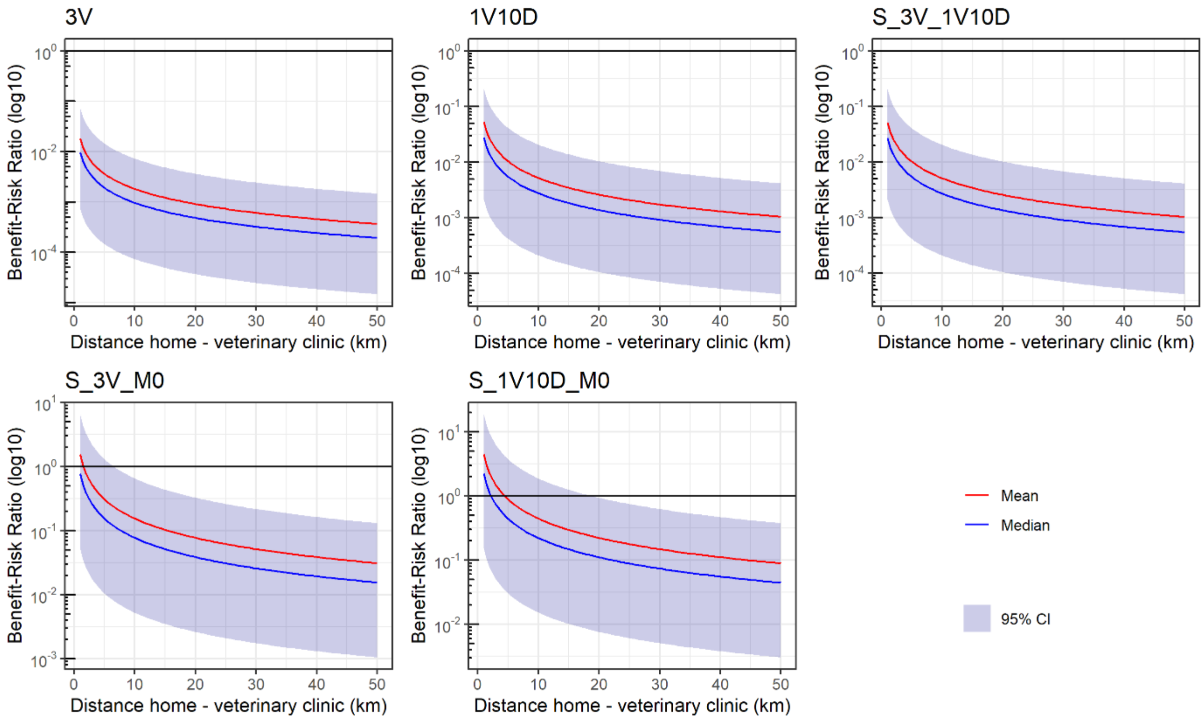

Figure C1. One-way sensitivity analyses on the home-veterinary clinic distance for dog bite surveillance protocol models. The distance considered corresponded to one journey between the home of the pet owner and a veterinary clinic. CI: confidence interval.

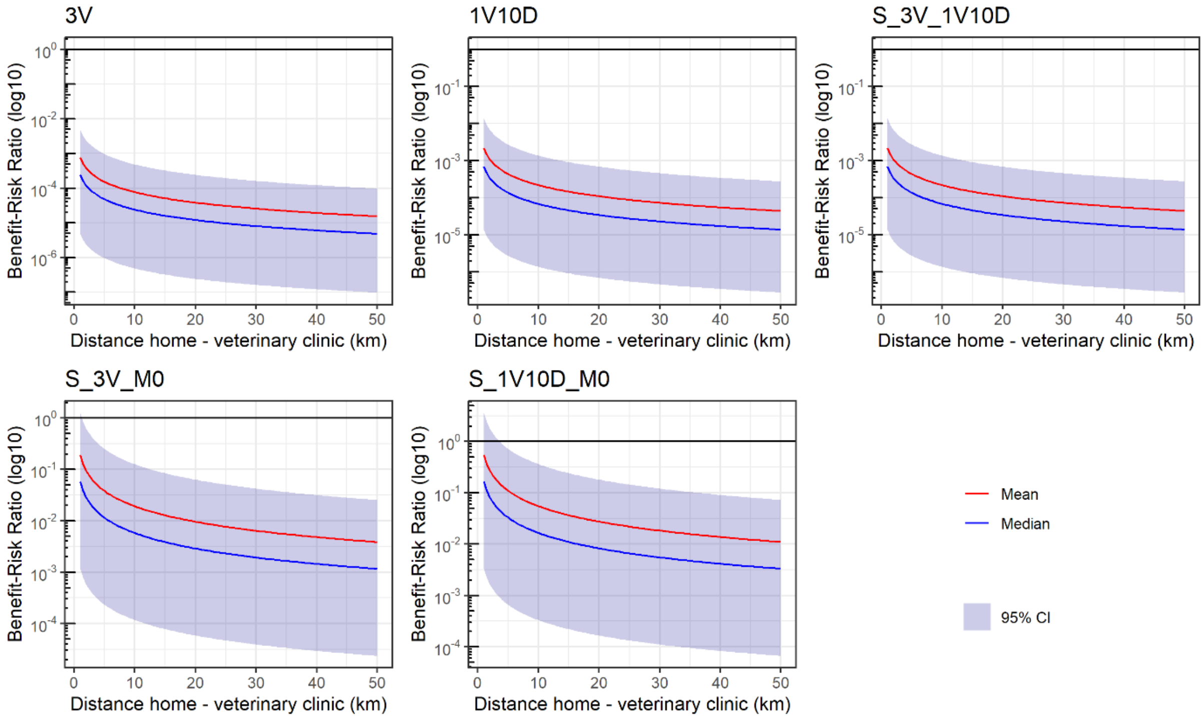

Figure C2. One-way sensitivity analyses on the home-veterinary distance clinic for cat bite surveillance protocol models. The distance considered corresponded to one journey between the home of the pet owner and a veterinary clinic. CI: confidence interval.

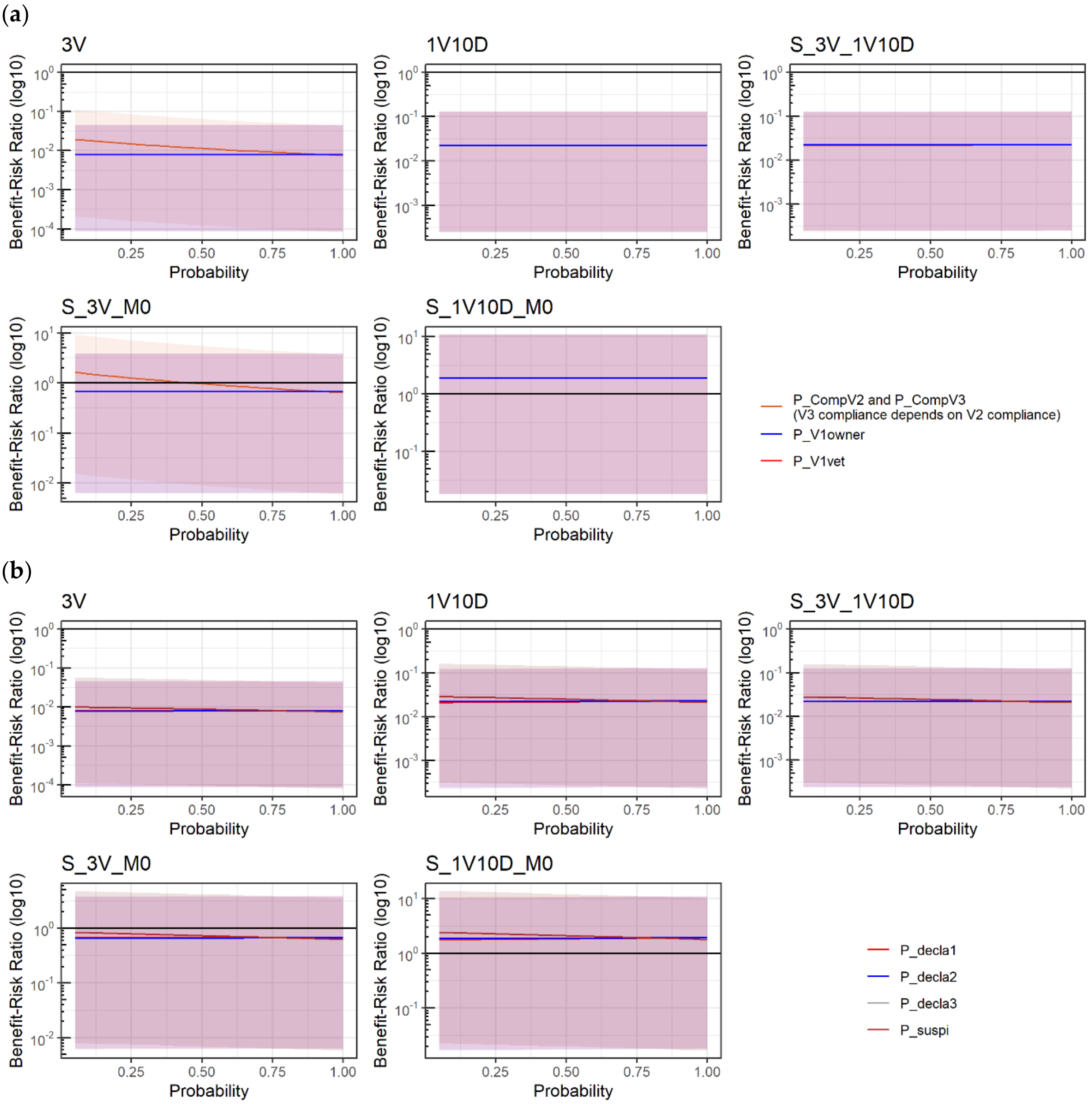

Figure C3. One-way sensitivity analyses on the compliance parameters for dog bite surveillance protocol models: (a) Veterinary visit compliance parameters; (b) Clinical sign declaration probability parameters. Lines represent the mean of the Benefit-Risk Ratio distributions and shaded areas the 95% confidence intervals

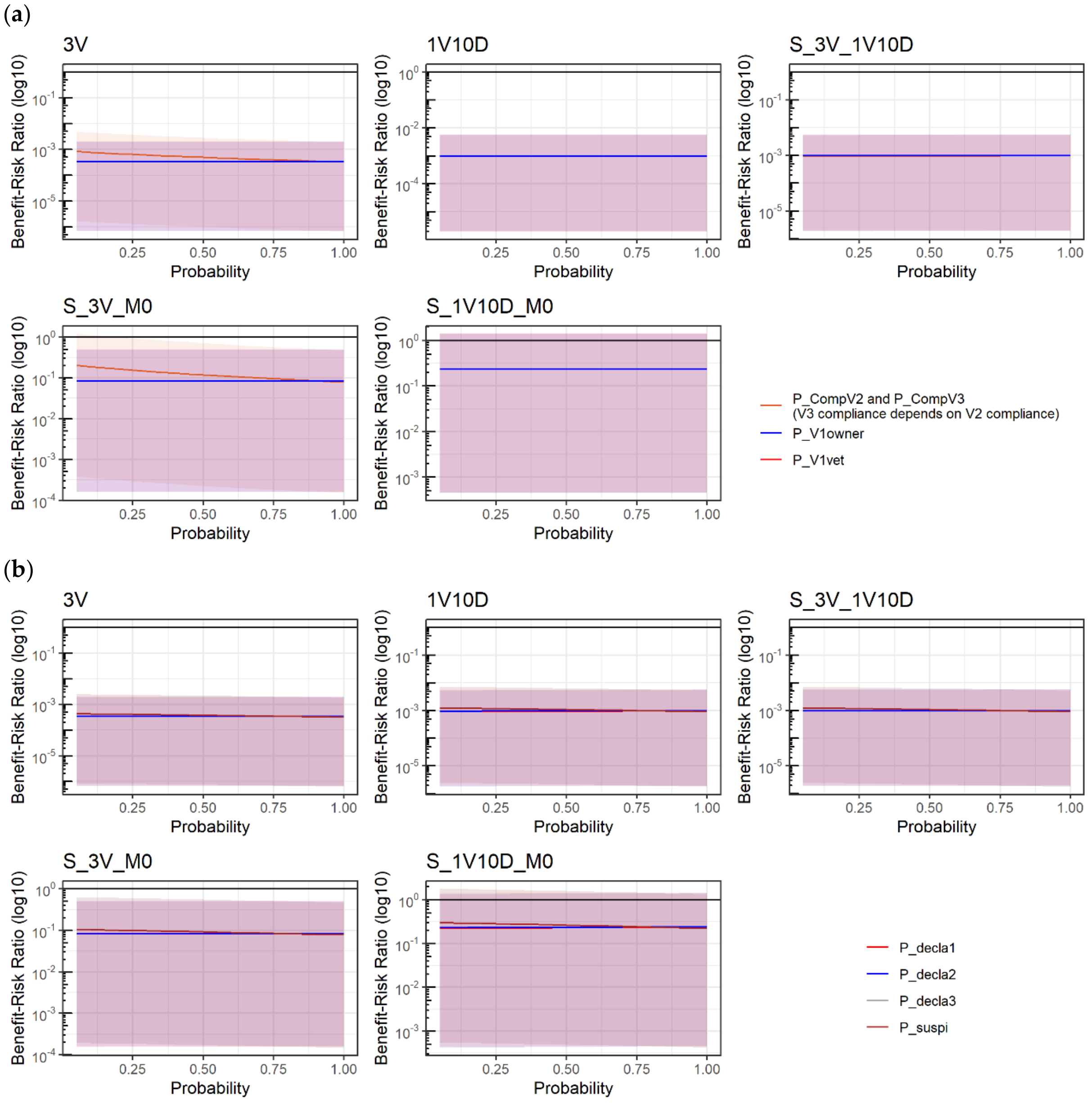

Figure C4. One-way sensitivity analyses on the compliance parameters for cat bite surveillance protocol models: (a) Veterinary visit compliance parameters; (b) Clinical sign declaration probability parameters. Lines represent the mean of the Benefit-Risk Ratio distributions and shaded areas the 95% credibility intervals.
